# Sotorasib resistance triggers epithelial-mesenchymal transition and activates AKT and P38-mediated signaling

**DOI:** 10.3389/fmolb.2025.1537523

**Published:** 2025-01-30

**Authors:** Raquel Arantes Megid, Guilherme Gomes Ribeiro, Izabela Natalia Faria Gomes, Ana Carolina Laus, Letícia Ferro Leal, Luciane Sussuchi da Silva, Abu-Bakr Adetayo Ariwoola, Josiane Mourão Dias, Rui Manuel Reis, Renato Jose da Silva-Oliveira

**Affiliations:** ^1^ Molecular Oncology Research Center, Barretos Cancer Hospital, São Paulo, Brazil; ^2^ Barretos School of Health Sciences, Dr. Paulo Prata-FACISB, São Paulo, Brazil; ^3^ Clinical Research Department, Barretos Cancer Hospital, São Paulo, Brazil; ^4^ Life and Health Sciences Research Institute (ICVS) Medical School, University of Minho, Braga, Portugal

**Keywords:** sotorasib, KRAS, NSCLC, sotorasib-resistant, combination therapy

## Abstract

**Background:**

The molecular non-genetic changes of resistance to sotorasib are currently uncertain. The aim of this study was to generate a sotorasib-resistant cell line via selective pressure and systematically examine the molecular and phenotypic alterations caused by resistance.

**Methods:**

Mutant NCI-H358 (KRAS^G12C^) were exposed to incremental doses (2–512 nM) of sotorasib. Then, resistant clones were separated by single-cell sorting. Proliferation was analyzed in real-time by xCELLigence; protein profiles were quantified by protein arrays; and mRNA expression profile was measured using the PanCancer Pathways panel by NanoString. *In silico* analyses were conducted from a database comprising patient-derived xenograft (PDX) models and cell lines resistant to sotorasib. AKT and p38. The synergistic effect of combining AKT, p38, and EGFR inhibitors was assessed using the SynergyFinder platform. Additionally, AKT and p38 genes were silenced using esiRNA.

**Results:**

Sotorasib-resistant H358-R cell line displayed markers of the mesenchymal-epithelial transition and loss of cell adhesion. Were identified 30 overexpressed genes in the resistance model, implicating in signaling pathways that leads to AKT activation and heightened protein expression levels of phosphorylated AKT and p38. To identify potential therapeutic strategies for overcoming sotorasib resistance, we investigated the combination of AKT and p38 inhibitors. Notably, combined inhibition of AKT (MK2206) and p38 (adezmapimod) restored sensitivity to sotorasib in resistant cell lines, as did silencing AKT expression.

**Conclusion:**

These findings underscore the importance of adaptive mechanisms in sotorasib resistance in NSCLC cells contributing by EMT activation and demonstrates synergic combination with AKT and p38 inhibitors to restore sotorasib sensitivity in KRAS^G12C^ cells.

## 1 Introduction

*KRAS* (Kirsten rat sarcoma two viral oncogene homolog) stands out as one of the most prevalent oncogenes across various human cancers (Prior et al., 2020). Despite robust evidence implicating oncogenic *KRAS* in tumorigenesis, endeavors to target mutant KRAS have encountered significant obstacles over the years (McCormick, 2016). However, recent advancements have emerged in the realm of therapeutics directed at the KRAS^G12C^ mutation. The discovery of a pocket situated in the switch-II region of KRAS has sparked interest in synthesizing compounds that can stably occupy this site and selectively target codon 12 in KRAS^G12C^ (Ostrem et al., 2013). Mechanistically, these agents exhibit the ability to stabilize KRAS in its inactive GDP-bound conformation, leading to the inhibition of downstream signaling pathways (Skoulidis et al., 2021). Furthermore, they induce stabilization of the guanosine diphosphate-bound (GDP-bound) state of the oncoprotein and, to some extent, prevents the binding of the RAF effector protein (Canon et al., 2019).

In patients diagnosed with advanced non-small cell lung cancer (NSCLC) carrying the KRAS^G12C^ mutation, sotorasib (AMG510 from AMGEN Inc.) and adagrasib (MRTX849 from MIRATY Inc.), both KRAS mutant specific G12C inhibitors, have exhibited significant efficacy with objective response rates of 37% and 45%, and disease control rates of 81% and 96%, respectively (Dhillon, 2023). Notably, sotorasib obtained FDA approval for the treatment of advanced KRAS^G12C^ NSCLC in patients who have previously undergone at least one systemic therapy, according to CodeBreaK100 phase I/II clinical trial (Blair, 2021). Subsequently, adagrasib was granted accelerated approval, bolstered by compelling evidence derived from the KRYSTAL phase I/II trial (Canon et al., 2019).

Although studies investigating KRASG12C inhibitors showed promising results, some patients exhibit unresponsiveness to these treatments, limited long-term efficacy, and disease recurrence. This observation indicates that intrinsic or acquired resistance represents an inevitable barrier for targeted molecular therapies. The primary mechanisms underlying the development of acquired resistance to targeted therapies include i) the acquisition of secondary mutations, ii) activation of bypass pathways, and iii) histological transformation of the tumor (Camidge et al., 2014).

The mechanisms behind acquired resistance to KRASG12C inhibitors are not fully understood. The mechanisms behind acquired resistance to KRASG12C inhibitors are not fully understood. Acquired resistance involves a complex interplay of both non-genetic and genetic changes, along with adaptive alterations. Understanding these mechanisms necessitates a comprehensive molecular analysis. This critical necessity underscores the importance of devising new strategies to enhance the efficacy of KRASG12C inhibitors while also addressing resistance.

*In vitro* cellular models play a pivotal role in comprehending the resistance phenotype of cancer treatment (Rosa et al., 2014). Consequently, our group developed a protocol for generating a resistant cell line, employing a selective pressure approach to obtained sotorasib-resistant clones. This method allowed us to discern molecular distinctions within the resistant cell population. In this study, we elucidated molecular alterations in sotorasib resistant cells by differential gene and protein expression analyses, with a focus on adaptive phenotypic, and molecular mechanisms associated with acquired resistance. Subsequently, we conceptualize combination therapies that can enhance the therapeutic efficacy of KRAS^G12C^ drugs and restore sensitivity in non-small cell lung cancer (NSCLC) cell lines with sotorasib-acquire resistance.

## 2 Materials and methods

### 2.1 Cell lines and reagents

*KRAS* ^G12C^ mutant H358 (RRID: CVCL_1559) and wild type *KRAS* H292 (RRID: CVCL_0455) cancer cell lines were used in this study, from the Cell Bank of the Barretos Cancer Hospital. Cell lines were maintained in RPMI 1640 complete medium, containing 10% fetal bovine serum (FBS), 2 mM glutamine, and 1% penicilin/streptomycin. Cells were incubated in a humidified atmosphere of 5% CO_2_ at 37°C. Cell culture reagents were purchased from Sigma-Aldrich (St. Louis, MO, United States), and to avoid the misidentified and/or cross-contamination, cell lines were authenticated by STR analysis (Silva-Oliveira et al., 2016). Cultures pellets were regularly tested for *mycoplasma* contamination by MycoAlert™ PLUS *Mycoplasma* Detection Kit (Lonza, Walkersville, MD, United States). All experiments were conducted in biological and experimental triplicates.

### 2.2 Pharmacological agents

Sotorasib anti-KRAS (G12C) (Cat. No. S8830), afatinib anti-Pan-HER (Cat. No. S2111), MK2206 anti-AKT (Cat. No. S1078) and adezmapimod anti-p38 (Cat. No S1076) inhibitors were purchased from Selleck Chemicals (Houston, TX, United States). All drugs were diluted in DMSO at 10 mM and stored at −20°C for future use. In all experiments, DMSO was used as control vehicles at a final concentration of 1% (v/v).

### 2.3 Sotorasib resistance model, single cell sorting and selection of resistant clones

A selective pressure model previously developed by our research group was used to establish resistance of the H358 cell line (Gomes et al., 2022). H358 cells were subjected to multiple cycles of sotorasib treatment (2–512 nM for 72 h), with varying recovery periods between cycles.

Isolation of sotorasib-resistant clones were performed by cell sorting with a BD FACSMelody™ Cell Sorter cytometer (BD Biosciences) (RRID:SCR_023209). After aseptic protocol, 384 cells were isolated in single wells and incubated under optimal culture conditions for individual cell clonogenic development. After recovery and 70%–80% of confluence, cells were re-exposed to the highest incremental dose of sotorasib (512 nM) for resistance phenotype validation. We established the following criteria for selection resistant clones to sotorasib: i) IC_50_ values greater than 1,000 nM and ii) persistent RAS-GTP activity of the resistant clones.

### 2.4 Cellular viability assay

Cell viability was assessed 72 h after drug treatments, using the colorimetric Presto Blue Assay (Thermo Scientific, Finland), according to the manufacturer’s instructions. For sotorasib cytotoxicity evaluation, 5 × 10^3^ cells were seeded in 96 well plates in complete RPMI allowing overnight adherence. Subsequently, cells were treated with increased sotorasib concentrations, and the fluorescence was measured using Varioskan Microplate Reader (Thermo Scientific, Finland), at 560/590 nm (excitation/emission). The results were normalized to DMSO control values. IC_50_ values were calculated using GraphPad Prism software (Version 9.0) (RRID:SCR_002798) by evaluating a nonlinear regression curve.

### 2.5 Ras-GTP form detection assay

Endogenous levels of active Ras (RAS-GTP) in the H358 parental and resistant clones were determined by Active Ras Detection Kit (Cell Signaling Technology, Inc), following the manufacturer’s instructions. A total of 7 × 10^6^ cells were lysed and 500 μL at 1 mg/mL of the protein fraction was incubated into glutathione resin with GST-Raf1-RBD. This mix was centrifuged on purification columns for RAS-GTP separation and subsequently elution. Thereafter, 20 μg of these samples were analyzed and quantified by western blotting methodology using a Ras mouse mAb.

### 2.6 Western blot and reverse phase protein arrays (RPPA)

Protein lysates of both lung cancer cell H358-P parental and resistant clones were used to perform western blot analysis in all steps of this study. Briefly, cells were rinsed in DPBS and after lysed in lysis buffer following our group protocol (da Silva-Oliveira et al., 2022). The primary antibodies were diluted in BSA 5% solution at 1:1000. Membranes were incubated with anti-rabbit (#7074) or anti-mouse (#7076) secondary antibodies at a dilution of 1:5000. Chemiluminescent signals were detected by ECL in the automatic ImageQuant mini LAS4000 (GE Healthcare). For further details regarding the antibodies used, please refer to Supplementary Table S1. Protein array Human Phospho-Mitogen-activated (ARY002B; R&D Systems, MN) and Human Phospho-RTK Array (ARY001B; R&D Systems, MN) were conducted according to the manufacturers’ instructions. A total of 1,000 ug/mL of protein lysate was incubated in the array membrane, and an immune dot signal was detected by ECL in automatic ImageQuant mini LAS4000 (GE Healthcare) (RRID:SCR_014246).

### 2.7 RNA isolation

RNA was isolated from H358-P (parental) and H358-R (resistant) clone cell lines using RecoverAll Total Nucleic Acid Isolation kit (Invitrogen), according to manufacturer’s recommendations. Samples were quantified by NanoDrop 2000 System (Thermo Scientific) (RRID:SCR_018042) and Qubit 2.0 Fluorometer (Life Technologies) (RRID:SCR_020553) and stored at −80°C for the further analysis.

### 2.8 mRNA expression profiling by NanoString

mRNA expression profile was evaluated for both H358-P (parental) and H358-R (resistant) cells, using the nCounter PanCancer Pathways Panel, which includes 770 genes associated to 13 cancer-associated canonical pathways related to basic cancer biology (Nanostring Technologies, United States) (RRID:SCR_023912) as previous described by our research team (Ge et al., 2020; Waggott et al., 2012). Briefly, 100 ng of total RNA (quantified by Qubit Fluorometric System) in triplicate were hybridized for 21 h at 65°C with Capture and Reporter probe pools, followed by purification and RNA/probe complex immobilization in the Nanostring PrepStation (Nanostring Technologies, United States) and cartridge scanning in the Digital Analyzer (Nanostring Technologies, United States), according to the manufacturer’s protocol, using 280 field-of-views (FOVs). Raw data were pre-processed by the nSolver Analysis Software v4.0® (NanoString Technologies) and normalized by the NanoStringNorm package (v1.2.1.1) in the R statistical environment (v3.6.3) employing normalization by housekeeping genes. The normalized log2-converted mRNA expression values were used for subsequent data analysis. Genes with fold change (FC) ≥ ± 1.5 and p < 0.05 were considered significant. Heatmap with hierarchical clustering of differentially expressed mRNA was built in the ComplexHeatmap package (v2.0.0). In silico interaction network analysis using these upregulated or downregulated key genes in H358-R (resistant) cells was conducted by STRING database (Version: 11.5, https://string-db.org); and enrichment analysis was conducted in web application ShinyGO: a graphical gene-set enrichment tool.

### 2.9 Cellular migration, invasion and adhesion assays

Cell migration was measured using inserts without Matrigel, and the detection of invasion was performed using the Invasion Chamber Kit (BD Biosciences, United States), following the manufacturer’s instructions. A total of 1.0 × 10^6^ cells were seeded into 24-well transwell inserts, in RPMI (serum-free). RPMI 10% FBS was used as a chemoattractant. After 48 h, the insert membrane was fixed with iced methanol and stained with hematoxylin/eosin. photomicrographs of the membranes were obtained under a ×40 magnification microscope, and the number of cells was counted by ImageJ software. The results were expressed as the mean percentage relative to the DMSO control (considered as 100% of invasion).

To assess cellular adhesion, the 96-well plate was coated for 24 h with a solution containing PBS, BSA (bovine serum albumin, 10 μg/mL, Sigma-Aldrich), Matrigel® (1:10 in PBS). After 24 h, the excess liquid was removed, and cell plates were incubated with 100 μL/well of 0.1% BSA for 2 h and washed with PBS. A total of 5.0 × 10^3^ cells were seeded and incubated at 37°C in a 5% CO_2_ humidified atmosphere for 2 h. Non-adherent cells were rinsed with PBS solution. Adhered cells were fixed with 10% of trichloroacetic acid (TCA), then stained with crystal violet solution (0.5%) and dissolved in 10% of acetic acid solution to absorbance detection at 590 nm using Varioskan Microplate Reader (Thermo Scientific, Finland). The absorbance values of the samples were plotted in GraphPad Prism software (Version 9.0).

### 2.10 Immunocytochemistry (ICC) detection

Cells were seeded in a chamber slide with eight wells on a glass slide allowing overnight adherence, and then fixed with 4% paraformaldehyde in DPBS for 5 min. After the fixation step, cells were permeabilized with Triton X-100 0.5% in DPBS for 4 min. Protein blocking was performed with Lab Vision™ UltraVision™ (Thermo Scientific), according to the manufacturer’s protocol. Primary antibodies ready to use vimentin (mAb #5741) (RRID: AB_10695459), E-caderin (#3195) (RRID: AB_2291471), Lef-1 (#2230) (RRID: AB_823558), pP38MAPK (Thr180/Tyr182) (#4511) (AB_2315112), pAKT-Ser473 (#4060) (RRID: AB_2315049) and pEGFR (#3777) (RRID: AB_2096270) were incubated overnight. After the incubation, biotinylated goat polyvalent antibody was added for 10 min, followed by washing and subsequently incubated with streptavidin peroxidase. The slides were stained with DAB chromogen and counterstaining with hematoxylin. Finally, the cells were photographed using the optical microscope Olympus XT01 at ×100 magnification.

### 2.11 Real-time cell proliferation analysis by xCELLigence®

Proliferation rates from H358-P (parental) and H358-R (resistant) cell lines were measured by xCELLigence systems analysis (Agilent Technologies, Inc) (RRID:SCR_019571), according to manufacturer’s instructions. Initially, 7.0 × 10^3^ cells were plated into an E-plate (Agilent Technologies, Inc) and were cultivated in complete RPMI medium for 96 h. After, 512 nM of sotorasib was added and the cell index and proliferation rates were calculated during the complete experiment using a specific RCCA® software analysis.

### 2.12 Real-time quantitative PCR

Real-time quantitative PCR (RT-qPCR) from H358-P (parental) and H358-R (resistant) cell lines were performed for specific genes linked to epithelial-mesenchymal transition (EMT), migration, and invasion using the GoTaq® DNA Polymerase system (Promega) according to manufacturer’s instructions. Real-time PCR was performed using a StepOne Plus instrument (Life Technologies, Carlsbad, CA, United States). PCR conditions were 95°C for 2 min to activate DNA polymerase, followed by 40 cycles at 94°C for 15 s and specific annealing temperature of each primer (Supplementary Table S2). The difference in cycle threshold value (Ct) of H358 parental *versus* internal control (^Δ^Ct) was used to determine gene expression in the H358 sotarasib-resistant cell.

### 2.13 In silico RNA-Seq analysis

We conducted an *in silico* analysis using RNA-Seq data from NCBI’s Gene Expression Omnibus database (GEO) (RRID:SCR_005012). Data from sotorasib-resistant cells and control (GSE204752), and resistant human PDX model (GSE204753) are used to correlate the molecular findings of the cell resistance model and clinical findings proposed in this study. The GEO2R application was utilized to compare two or more groups of samples to identify genes differentially expressed across experimental conditions. P-values were adjusted using the Benjamini & Hochberg method (false discovery rate), with a significance level cut-off set at 0.005 (Barrett et al., 2013), according to web-application available at https://www.ncbi.nlm.nih.gov/geo/info/geo2r.html#references. After obtaining the complete raw data, P-values were calculated using an unpaired *t-test*, and differentially expressed genes were identified using GraphPad Prism software (version 9.0). Bar graphs are presented as mean ± SEM.

### 2.14 Drugs combination index (C.I)

To determine the optimal therapeutic combination that is effective against the sotarasib resistance phenotype, we used a dilution matrix containing five different concentrations of MK2206 (anti-AKT) and adezmapimod (anti-p38) drugs, both diluted in DMSO. After 72 h, the cell viability was determined. Subsequently, the raw data was analyzed on the SynergyFinder (RRID:SCR_019318) web-application available at https://synergyfinder.fimm.fi/ by three reference methods (ZIP, Bliss and Loewe). Synergism between two drugs was classified according to this score: values below −10: the interaction between two drugs is probably antagonistic; values from −10 to 10: the interaction between two drugs is probably additive; values greater than 10: the interaction between two drugs is probably synergistic.

### 2.15 mRNA silencing

AKT and p38 silencing was performed using MISSION® esiRNA (Eupheria Biotech, EA) (Sharaf et al., 2024). Each siRNA duplex was transfected into H358-R (resistant) cells using Lipofectamine3000 (Life Technologies), according to the manufacturer’s protocol. H358-R cells were plated in 6-well plates at a density of 3.5 × 10⁵ cells/mL in DMEM-10% and allowed to adhere overnight. Cells were then transfected with 28 pmol of target-specific esiRNAs against AKT (isoforms 1 to 3), p38 or RLUC (used as control) mRNAs in reduced serum Opti-MEM media (31985062 - Gibco, Invitrogen) for 6 h. Lastly, AKT and p38 protein expression levels were measured 48 h post-transfection by western blot, and viability was analyzed using the MTS and Apotox-Glo triplex assays (Promega, United States).

### 2.16 3D spheroids culture

The impact of silencing the highly expressed AKT and p38 genes in the H358-R (resistant) cell line was assessed using a 3D model. Specifically, 1.0 × 10^4^ previously silenced cells were seeded in 96-well ultra-low attachment plates (#174929, Thermo Scientific, Finland), centrifuged at 300 g for 10 min, and incubated for 48 h at 37°C. After incubation, the spheroids were treated with varying concentrations of sotorasib. The following day, caspase 3/7 activity was evaluated via fluorescence microscopy using the CellEvent™ Caspase-3/7 Green Detection Reagent (#C10423, Invitrogen). Spheroid images were subsequently captured at ×10 magnification using an Olympus IX71 microscope.

## 3 Results

### 3.1 Development and characterization of resistance to sotorasib model

**FIGURE 1:**
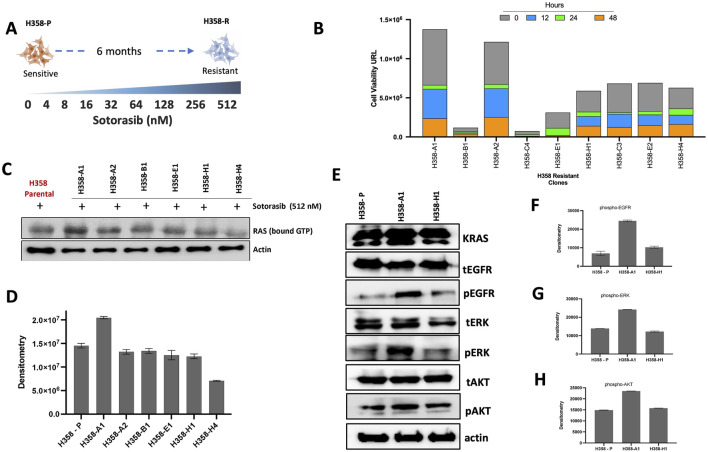
Establishment and characterization of cell lines resistant to sotorasib. **(A)** Scheme of the incremental sotorasib resistance model. H358-P (paental) and H358-R (resistant); **(B)** Viability analyses of sotorasib-resistant clones, after single cell sorting, 0–48 h of sotorasib exposure are represented by bar graphs; **(C)** RAS GTP-bound profile of resistant clones, after sotorasib exposure, detected by Western blot; **(D)** Densitometric analyses of RAS GTP-bound are represented by bar graphs; **(E)** Total and phosphorylated protein profile resistant clones by Western blot; **(F)** Densitometric analyses of phospho-EGFR; **(G)** Densitometric analyses of phospho-ERK; **(H)** Densitometric analyses of phospho-AKT.

The sotorasib-resistant model was created by subjecting the sensitive H358 cell line to a series of incremental sotorasib doses over time. The initial IC_50_ value for the sensitive H358 cell line was 4.02 ± 0.1 nM after 72 h. A total of eight incremental concentration steps were required to obtain the sotorasib resistance phenotype reaching the maximum dose of 512 nM (Figure 1A). For isolation of cells with distinct resistance patterns, the resistant cells were subjected to single cell sorting, which resulted in a total of 33 clones. These clones were re-treated with 512 nM sotorasib, and only nine clones (A1, B1, A2, C3, C4, E1, E2, H1, H4) exhibited resistance at this stage displaying more than 150 times higher IC_50_ values (665.5 to >1000 nM) (Supplementary Table S3) when compared with H358 sensitive cell line. H358-A1 and H358-A2 clones demonstrated higher cell viability, which indicate lower effect of sotorasib at 0, 12, 24 and 48 h (Figure 1B).

The levels of active (GTP-bound) RAS were greater in H358-A1 resistant clone after sotorasib exposure, compared to the other selected clones (Figures 1C, D). H358-A1, showed an increase of pEGFR and pERK levels, compared to the protein expression profile of the H358-P (parental) cell line (Figures 1E–H), while the H358-H1 clone displayed a similar profile to the parental sensitive cell line. Given its overall reduced response to sotorasib, H358-A1 was chosen for subsequent analysis and named H358-R (resistant).

### 3.2 Phenotype pattern changes in sotorasib resistant cells

**FIGURE 2:**
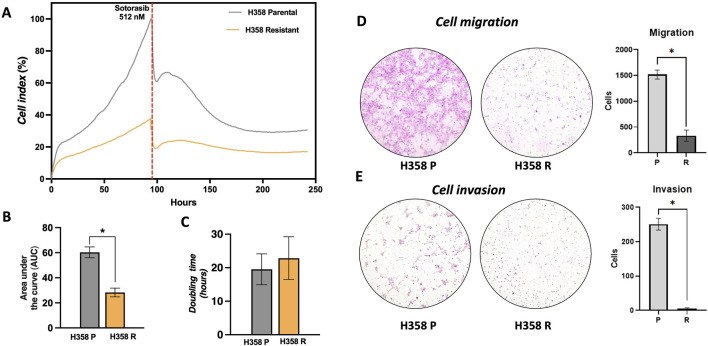
Phenotype changes in sotorasib resistant cells. **(A)** Real-time cell analysis showing the cell index (C.I) of H358-P (parental) and H358-R (resistant) cells following sotorasib exposure, measured over a time course of 250 h; **(B)** Area under the curve (AUC), of H358-P and H358-R cells are represented by bar graphs at 100 h; **(C)** Double time of H358-P and H358-R cells are represented by bar graphs at 100 h; **(D)** Photomicrography at ×40 magnification from cell migration of H358-P parental and H358-R cells, and bar graph represents the number of cells counted; **(E)** Photomicrography at ×40 magnification from cell invasion of H358-P (parental) and H358-R (resistant) cells, and bar graph represents the number of cells counted.

Sotorasib-resistant cells exhibited a significant decrease in the overall proliferation compared to the parental H358-P cell line (Figure 2A). After 96 h, the proliferative cell index of the H358-R cells was 37.8%, compared to parental H358-P cell index (100%). When sotorasib was added (512 nM) to the H358 parental cell line, it resulted in a reduction of 70% in the cell index. However, the resistant H358-R cell population exhibited a less pronounced cell index, with levels no greater than 21.5% (Figure 2A). In accordance with these findings, significantly decreased in the area under the curve and increased doubling time of the H358-R cell line, when compared with H358-P cell line (Figures 2B, C). It was estimated that approximately 23 h were required for H358-R to double its population size, whereas only 19 h were required for H358-P (Figure 2C). The migration potential of the H538-R cell line was altered due to sotorasib resistance, resulting in lower migration and invasion rates, compared to the parental H358 cell line (Figures 2D, E).

### 3.3 Sotorasib resistance promotes epithelial-mesenchymal transition

**FIGURE 3:**
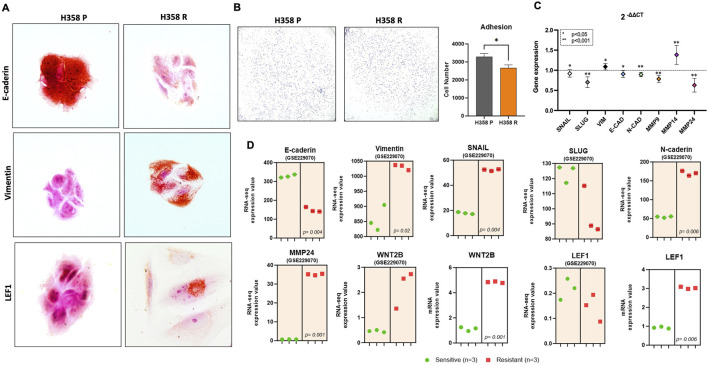
Molecular markers of epithelial mesenchymal transition. **(A)** Photomicrography at ×100 magnification from adhesion cells and the bar graph represents the number of adhered cells; **(B)** EMT markers expression by RT-PCR. The data are presented in fold-change in comparison with H358-P (parental) cell line; **(C)** Photomicrography at ×100 magnification from immunohistochemical staining for comparative EMT markers among the H358 parental and H358-R (resistant) cells; **(D)** Differential gene expression from GSE229070 (graph with orange area) and H358-R sotorasib-resistant cell line (graphic without colored area).

Immunocytochemistry photomicrographs of both sotorasib sensitive (H358-P) and resistant (H358-R) cells were conducted to investigate the protein levels in cellular context and revealed a noteworthy upregulation in the expression levels of *Vimentin* and the *LEF1* transcription factor, in the H358-R cell line, concurrently with a decrease in *E-Cadherin* levels (Figure 3A). Cell adhesion assays revealed that the H358-R (resistant) cell line displayed decreased cell-protein binding compared to the parental H358-P cell line (Figure 3B). Furthermore, gene expression analyses by RT-PCR of targeting biomarkers associated with the epithelial phenotype demonstrated a significant decrease in *SNAIL, SLUG*, *E-Cadherin*, *N-Cadherin*, *MMP-9* e *MMP-24* expression. To validate our results, we explored the dataset (GSE229070) derived from another sotorasib-resistance model, and we observed a similar result to *E-Cadherin* and *SLUG* (Figure 3D). In addition, both sotorasib resistance models exhibited elevated expression levels of *WNT2B* and *LEF1* (Figure 3D).

### 3.4 The differential gene expression profile of the sotorasib resistance model

**FIGURE 4:**
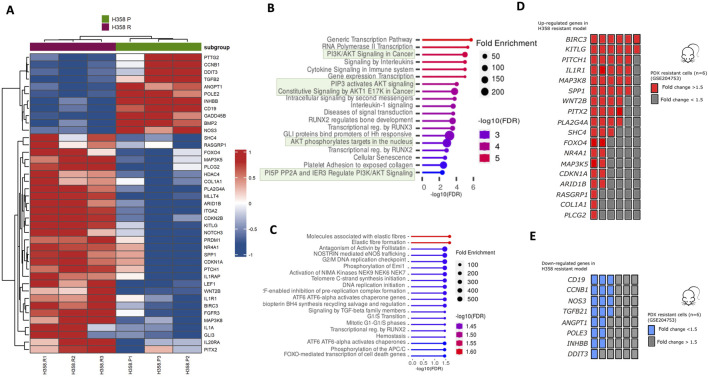
Differential expression gene set and activation of signaling pathways in the sotorasib-resistant model. **(A)** Heatmap of genes altered in H358-R (resistant) compared with H358-P (parental) cells from nCounter PanCancer Pathways Panel; **(B, C)** Bubble plot of REACTOME pathway enrichment analysis from 41 differentially expressed genes in H358-R (resistant) cell line compared with H358-P (parental); Upper bubble graph corresponds to overexpressed genes (n = 30) and lower to downregulated genes (n = 11); **(D)** Number of patient-derived xenograft (PDX) resistant cells from GSE204753 exhibiting a comparable overexpression pattern of genes as identified in our sotorasib-resistant cell line, H358-R (resistant); **(E)** Number of patient-derived xenograft (PDX) resistant cells from GSE204753 exhibiting a comparable overexpression pattern of genes as identified in our sotorasib-resistant cell line, H358-R.

Using the Nanostring platform, we compared the mRNA expression profile of sotorasib-resistant H358-R (resistant) with H358-P (parental) cells. Further information regarding this analysis can be found in the Supplementary Information section. We found 41 differentially expressed genes after acquired resistance to sotarasib considering 1.5 of fold-change (Figure 4A; Supplementary Table S4). Of note, upregulation of *GLI3*, *NR4A1*, *PTCH1*, *SHC4*, *IL1A*, *FOXO4*, *BIRC3*, *LEF1*, *RASGRP1*, *PITX2*, *IL20RA* and *WNT2*, exhibited more than 2.0-folds in the sotorasib-resistant H358-R cell line (Figure 4A; Supplementary Table S4). We also observed downregulation (less than −2.0-folds) of the genes *ANGPT1*, *CD19*, *NOS3*, *TGFB2*, and *CCNB1* in the H358-R cell line (Figure 4A; Supplementary Table S4). The functional enrichments suggested potential interactions with five pathways mediated by AKT signaling such as PI3K/AKT signaling in cancer, PIP3 activates AKT signaling, AKT phosphorylates targets in the nucleus and PI5P PPA and IER3 regulate PI3K/AKT signaling (Figure 4B). Using the GSE204753 dataset from transcriptomic analysis of sotorasib-resistant patient-derived xenograft (PDX) lung tumor cells and compared to our resistant model, we identified a consistent overexpression of genes *BIRC3*, *KITLG*, *PITCH1*, *IL1R1*, *MAP3K8* and *SPP1*. Notably, these genes demonstrated increased expression in at least four out of six transcriptome analyses conducted on sotorasib-resistant PDX samples (Figure 4C). Among the downregulated genes identified in the sotorasib-resistant H358-R cell line, the functional enrichments suggested an interaction with five pathways involved in cell cycle control, such as initiating DNA replication or cell cycle checkpoints (Figure 4D). In addition, *CD19*, *CCNB1*, *NOS3* and *TGFB1* genes exhibited decreased expression in at least three of six transcriptome analyses of sotorasib-resistant patient-derived xenograft (PDX) (Figure 4E).

### 3.5 The development of sotorasib resistance led to an increased activation of MAPK and RTK signaling pathways

**FIGURE 5:**
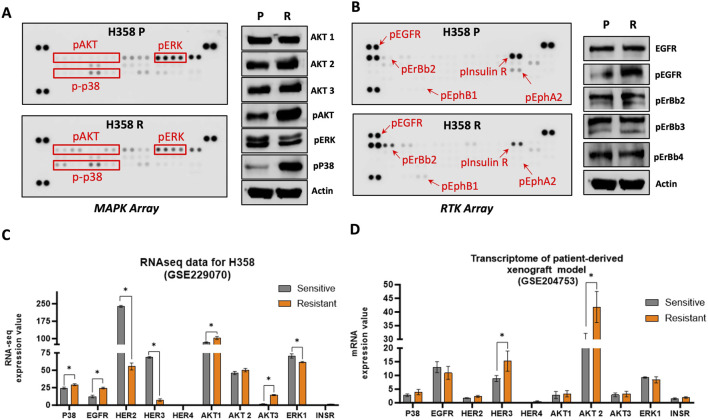
Activation of MAPK and RTK signaling pathways in sotorasib resistant cell. **(A)** Representative pictures of phospho-MAPK arrays for H358-P (parental) and H358-R (resistant) cell lines. **(B)** Representative pictures of phospho-RTK arrays for H358-P (parental) and H358-R (resistant) cell lines. For both arrays protein is represented in duplicate in the arrays (two spots side by side) and four pairs of phosphotyrosine positive controls in the corners of each array. Arrows or red squares represent the differential protein levels. The protein expression analysis of the H358-R (resistant) cell line was normalized and compared to that of the H358-P (parental) cell line. **(C)** Bar graph representations of the differential expression profiles of genes from GES229070 dataset. **(D)** Bar graph representations of the differential expression profiles of genes from GSE204753 dataset. *p < 0.05.

To characterize the difference in protein profile of the parental H358-P and resistant H358-P cell lines, we initially investigated MAPK expression, using a Human Phospho-MAPK Protein Kinase assay. H358-R cells exhibited increased levels of phosphorylated AKT1, AKT2 and AKT3 isoforms (Figure 5A; Supplementary Figure 1). We also observed a significant increase in the expression levels of phosphorylated p38 in H358-R. This finding was further validated through western blot analysis and Immunocytochemistry (Figure 5A; Supplementary Figure 2). The expression levels of ERK were found to be slightly decreased in the sotorasib-resistant H358-R cells. However, no significant differences were identified upon western blot validation (Figure 5A).

The expression of receptor tyrosine kinases (RTKs) under non-treatment conditions was evaluated using a Human Phospho-RTK Protein Kinase assay. We observed an increase in pEGFR, pHER2, and pEphB1 in the sotorasib-resistant H358-R cell line (Figure 5B). Conversely, the phosphorylation levels of the Insulin receptor were significantly reduced in the H358-R (resistant) cell line (Figure 5B). In addition, immunocytochemical photomicrographs of sotorasib-sensitive and resistant cells confirmed the substantial increase in pEGFR protein levels (Supplementary Figure 2).

Confronting the GSE229070 dataset, revealed a significant upregulation in the expression of several genes, including *P38, EGFR*, *HER2*, *AKT1*, *AKT2*, *AKT3*, *ERK1*, and *INSR,* when compared with sotorasib sensitive cell line H358. These findings aligned with the protein alterations observed in our sotorasib-resistant model, with HER3 being the sole exception (Figure 5C). Furthermore, transcriptome data (GSE204753) obtained from a sotorasib-resistant patient-derived xenograft model demonstrated a notable increase in the expression, HER3, and AKT2, aligning with the findings in our sotorasib-resistant model (Figure 5D).

### 3.6 AKT and p38 inhibition restores sensitivity to sotorasib in resistant cell line models

**FIGURE 6:**
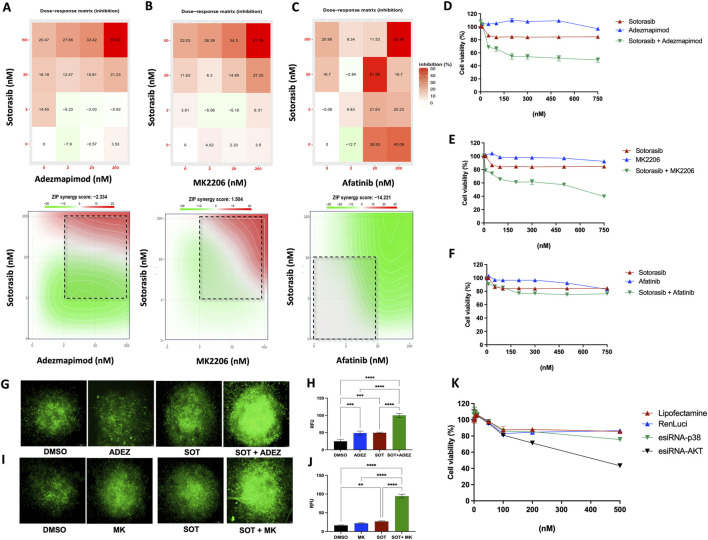
Restored sotorasib sensibility to H358-R (resistant) cells by therapy combination. **(A)** Combination dose-response matrix between sotorasib and adezmapimod (anti-p38); **(B)** Combination dose-response matrix between sotorasib and MK2206 (anti-AKT); **(C)** Combination dose-response matrix between sotorasib and afatinib (anti-EGFR). Heatmap represents the percentage of inhibition between drug concentrations and maps highlight synergistic and antagonistic dose regions in red and green colors, respectively. Dashed square indicated the most synergistic area; **(D)** Dose-response curve of the sotorasib-adezmapimod combination, 72 h post-treatment; **(E)** Dose-response curve of the sotorasib-MK2206 combination, 72 h post-treatment; **(F)** Dose-response curve of the sotorasib-afatinib combination, 72 h post-treatment; **(G)** Caspase 3/7 activity detection via fluorescence microscopy, 24 h post-treatment with sotorasib and adezmapimod; **(H)** Quantification of caspase 3/7 activity-mediated fluorescence, 24 h post-treatment with sotorasib and adezmapimod; **(I)** Caspase 3/7 activity detection via fluorescence microscopy, 24 h post-treatment with sotorasib and MK2206; **(J)** Quantification of caspase 3/7 activity-mediated fluorescence in 3D spheroids, 24 h post-treatment with sotorasib and MK2206. Spheroid images captured at ×10 magnification in an Olympus IX71 microscope; **(K)** Dose-response curve of the sotorasib combination with p38 or pAKT genetic silencing (mediated by RNA interference), 72 h post-treatment.

Pharmacological interactions were observed between the AKT inhibitor MK2206, the p38 inhibitor adezmapimod, and the pan-EGFR inhibitor afatinib when combined with sotorasib. Combination index analysis revealed a ZIP score of −2.33 for adezmapimod, 1.56 for MK2206, and -14.22 for afatinib in combination with sotorasib. Particularly, the combination of sotorasib and adezmapimod exhibited an additive effect, with inhibitory effects of 20.4% and 37.0% at the lowest and highest dosages, respectively (Figure 6A). Moreover, this additive effect persisted when the concentration range for both drugs was expanded (Figure 6D). As shown in Figure 6B, the combination of sotorasib and MK2206 exhibited a synergistic effect, with inhibition reaching 26.0% and 51.7% at the lowest and highest dosages, respectively. This synergistic effect was dose-dependent, peaking at approximately 60% inhibition at 750 nM (Figure 6E). Loewe-Bliss combination index analysis consistently classified the combination of AKT and p38 inhibitors as additive (CI = −0.57 to 0.33; Supplementary Table S5). In contrast, the combination of sotorasib and afatinib exhibited a pronounced antagonistic effect (CI < −10; Figure 6C; Supplementary Table S5). Notably, this drug combination did not significantly impact cell viability across the tested concentration range (Figure 6F).

To assess whether the observed combinatorial effect could be replicated in a 3D model, we measured Caspase 3/7 activity using immunofluorescence microscopy after treating cells with sotorasib combined with either adezmapimod or MK2206. As depicted in Figures 6G, H, 24 h post-treatment, the combination of sotorasib and adezmapimod led to an increase in caspase 3/7 activity compared to either drug alone. Moreover, the combination of sotorasib and MK2206 (Figures 6I, J) resulted in an even more pronounced increase in Caspase 3/7 activity.

To further validate the potential of AKT and p38 inhibition as a therapeutic strategy to restore sotorasib sensitivity in our resistant model, gene knockdown via RNA interference was performed. Subsequently, cells were treated with increasing concentrations of sotorasib, and their viability and protein levels were assessed. Western blot analysis confirmed the reduction in expression of both AKT and p38 after 48 h, which was maintained upon sotorasib treatment (Supplementary Figure 3). Consistent with our findings using pharmacological inhibition, combining AKT or p38 gene knockdown with increasing concentrations of sotorasib resulted in reduced viability of H358-R cells (Figure 6K).

## 4 Discussion

In recent years, selective small-molecule inhibitors targeting the G12C mutation have been identified, such as sotorasib (AMG510) and adagrasib (MRTX849). Based on results from the CodeBreak trial and KRYSTAL-1, sotorasib and adagrasib, respectively, have received FDA approval for treating patients with KRAS^G12C^ mutation advanced non-small cell lung cancer (NSCLC) who have experienced disease progression after prior chemotherapy and/or checkpoint inhibitors (Janne et al., 2022; Strickler et al., 2023). Nevertheless, the emergence of resistance to these inhibitors has raised substantial concerns (Awad et al., 2021). This accentuates the critical need for an impartial understanding of the mechanisms sustaining resistance. Clinical studies involving patients still demonstrated several limitations, primarily attributable to the small number of patients subjected to anti-KRAS therapy. The most comprehensive study conducted by Awad *et al.* detected the presence of a KRAS^Y96C^ mutation in one patient with NSCLC of 38 patients with KRAS^G12C^ treated with adagrasib who initially had stable disease for at least 12 weeks (Awad et al., 2021). Notably, this study highlighted a novel acquired secondary mutation, KRAS^Y96C^, within the switch II pocket where both adagrasib and sotorasib bind. This emphasizes the motivation behind our investigation into non-genetic adaptations facilitated by the reprogramming of oncogenic signaling pathways.

To better characterize the acquired resistance of sotorasib, we performed a model of incremental and prolonged exposure to sotorasib that lasted 6 months, until we obtained a heterogeneous population of resistant cells. Similar resistance acquisition models have already been conducted by other groups, despite the fixed concentration of sotorasib (Chan et al., 2023). However, the average exposure time to sotorasib in these studies was two (Chan, Chiou, Lee, Liu, Hsieh, Yang and Jeng, 2023) and nine (Zhao et al., 2021) months and the analysis was performed on the entire resistant cell population. To resolve this bias, we conducted the single cell sorting for resistant population to isolating and select the most resistant clone, based on undetectable IC_50_ values, inefficient GTP-bound RAS blockade, reactivation of the MAP kinase signaling pathway and high levels of phosphorylation of the EGFR receptor and ERK1/2.

In general, we detected a severe reduction in the proliferation, migration and adhesion competence of the H358-R cell line. This phenotype may be related to the fact that cells can adapt to targeted therapies, in which drugs interfere with the molecular signaling necessary for proliferation and survival. Activation of drug resistant mechanisms consume energy that would otherwise be available for invasion into non-cancerous tissues or proliferation and thus reduces the cell’s aptitude, also called cellular fitness. This fact may cause an increase of metabolic cost by drug resistance phenotypes and consequently suppressing migration, invasion and other cellular activities (Gatenby, 2009).

The H358-R (resistant) cell line overexpressed 30 genes in our cellular model of sotorasib resistance, with the *RasGRP1*, *IL20RA*, *WNT2B*, *LEF1*, and *PITX2* genes exhibiting the highest levels of expression. One of the *RasGRP* family’s members, Ras guanine nucleotide-releasing protein 1 (RasGRP1), is a guanine nucleotide exchange factor involved in Ras activation (Stone, 2006). It plays a role in several biological processes, notable among them being the facilitation of the Ras cycle that shifts the protein from its GDP-bound inactive form to its GTP-bound active form (Cullen and Lockyer, 2002). As a predictive biomarker for EGFR inhibitors in colorectal cancer (Gbenedio et al., 2019), *RasGRP1* has been linked to several cancer-related processes, such as hepatocarcinoma carcinogenesis (Zhang et al., 2024) and displaying an association with improved overall survival in breast cancer (Wang B. et al., 2018). Notably, this study establishes the first link between overexpression of the *RasGRP1* gene and the emergence of acquired resistance mechanisms to sotorasib.

Particularly, the expression levels of binding receptors and activated WNT pathways vary due to the diverse members of the WNT family, and they are closely linked to the occurrence and progression of non-small cell lung cancer (NSCLC) (You et al., 2002). Specifically, WNT2B protein levels in NSCLC patient tissues have demonstrated a significant increase compared with normal tissues and correlating with unfavorable patient outcomes (Huang et al., 2015), furthermore chronic treatment with sotorasib has been associated with WNT expression and activation of the WNT/β-catenin signaling pathway (Mohanty et al., 2023). No association with resistance to KRASG12C-specific inhibitors has been attributed to the WNT2B gene. However, *in silico* analysis revealed increased expression in patient-derived xenograft (PDX) resistant cells as well as *in vitro* models in agreement with the findings of our sotorasib resistance model. Notably, WNT2B overexpression promoted proliferation, colony formation and the EMT process in NSCLC cells with miR-577 overexpression and under basal conditions (Wang S. et al., 2018) and are directly regulated by the transcription factor PITX2, which was highly overexpressed in our sotorasib resistance model, along with *LEF1* gene, that also plays a key role in activating WNT/β-Catenin-mediated pathway and is portrayed in lung adenocarcinoma metastasis (Nguyen et al., 2009). It has been established that Wnt signaling plays a crucial role in EMT, and the Wnt/GSK-3β axis, specifically regulates the degradation of epithelial markers, including cadherins (Wu et al., 2017). The degradation of E-Cadherin, a key protein in adherence junctions, thereby contributing to the reduction in cellular adhesion, exactly as observed in our sotorasib resistance model, which indicates the reprogramming to a mesenchymal phenotype. In contrast, sotorasib resistance models constructed with lower doses and shorter durations showed a notable increase observed on the adhesion potential (Chan et al., 2023).

The process of epithelial-mesenchymal transition (EMT) represents a critical biological mechanism through which polarized epithelial cells, typically anchored to the basement membrane via their basal surface, undergo extensive biochemical transformations. These changes enable the cells to acquire a mesenchymal phenotype, characterized by increased migratory and invasive capacities, heightened resistance to apoptosis, and a significant upregulation in the production of extracellular matrix (ECM) components (Kalluri and Weinberg, 2009). Recent findings have revealed that the EMT status of circulating and disseminated tumor cells (CTCs/DTCs) plays a pivotal role in their metastatic potential. Interestingly, epithelial-like CTCs with minimal mesenchymal transition exhibited the highest capacity for forming lung metastases. In contrast, CTCs predominantly exhibiting mesenchymal characteristics displayed a significantly reduced ability to metastasize, underscoring the nuanced relationship between EMT phenotypes and metastatic efficiency (Jaiswal and Yadava, 2020). In the context of KRAS inhibition therapies, EMT emerges as a prominent mechanism driving acquired drug resistance, ultimately contributing to the failure of cancer treatment. This intricate phenomenon is orchestrated by epigenetic reprogramming and the activation of transcription factors intricately linked to EMT (Kalluri and Weinberg, 2009). A recent study indicates that non-genetic adaptations play a pivotal role, involving the rewiring of oncogenic signaling pathways, intricate reciprocal interactions between cancer cells and the tumor microenvironment (TME), and the dynamic phenotypic plasticity exemplified by EMT in adaptive resistance to KRAS-G12C inhibition (Ning et al., 2022). A study involving patients treated with sotorasib displaying partial or inefficient responses, unveiled multiple mechanisms of resistance to current KRAS-G12C inhibitors. The investigation emphasized the enrichment of clonal populations, underscoring a notable augmentation in EMT mechanisms across the various sub-clones analyzed (Tsai et al., 2022). Consistently, in our cellular model, we observed key features supplementary the activation of the EMT.

Epithelial-mesenchymal transition encompasses a series of phenotypic and molecular alterations observed both during normal developmental processes and in cancer progression, often correlating with poorer prognoses (Larue and Bellacosa, 2005). The relationship between the PI3K/AKT signaling axis has demonstrated the essential regulatory role of this pathway in EMT (Grille et al., 2003) and the AKT inhibition induced the expression of E-cadherin and beta-catenin, reduce that of Vimentin, restored their epithelial morphology of oral squamous cell carcinoma (Hong et al., 2009). AKT kinases, downstream of PI3K, serve as primary effectors of EMT signaling, with constitutively active AKT mutants shown to induce EMT in carcinoma cell lines (Fattahi et al., 2020). In our study, when compared to the H358-P (parental) cell line, the protein profile of the H358-R (resistant) exhibited a noteworthy increase in the expression of pAKT and p38, which were also detected by immunocytochemistry. Among the differentially expressed proteins in our model, we observed a notable upregulation of p38, which is associated with MAPK signaling what happens typically in mitogen response, environmental stresses, growth arrest, and apoptosis, engaging with various cytoplasmic and nuclear substrates (Canovas and Nebreda, 2021) and involvement of EMT (Ryu et al., 2019b). Activation of p38 are critical mediators in the processes of tumor cell invasion and metastasis (del Barco Barrantes and Nebreda 2012). Extensive research has demonstrated that the p38 signaling pathway plays a significant role in regulating epithelial-mesenchymal transition (EMT), a key event associated with enhanced tumor invasiveness and metastatic potential in lung adenocarcinoma (Zhang et al., 2017). Studies have further highlighted the involvement of p38 in modulating the expression of EMT-associated protein markers, such as E-cadherin and vimentin, (Tadokoro et al., 2016), Snail (Ryu et al., 2019a) and that also founded in our sotorasib resistant model.

In NSCLC cells harboring a *KRAS* mutation (G12C, Q61H and G12R), oncogenic *KRAS* can directly influence the activation of p38α, sustaining cell proliferation even in the presence of MEK inhibition (Sunaga et al., 2019). To date, no studies have linked elevated p38 protein expression to sotorasib resistance in NSCLC patients. Supplementarily, the combination of p38 inhibitors with sotorasib has not been previously explored. Our findings reveal a synergistic effect between sotorasib and the p38 inhibitor adezmapimod, leading to enhanced cell inhibition. This is supported by significant cell viability reduction following p38 knockdown and increased Caspase 3/7-mediated apoptosis in 3D models.

In tandem, the enrichment analysis of signaling pathways conducted with the set of genes overexpressed in the resistant cell line, revealed a positive modulation of pathways mediated by AKT activation, which demonstrates an involvement in AKT signaling pathway in response to sotorasib resistance. Overactivation of AKT is a common molecular feature in various human malignancies, including lung cancer (Guo et al., 2013) and gastric cancer (Kim et al., 2017). In certain cases, targeting direct activators such as TBL2 and PRMT5 has been explored as a potential therapeutic strategy for breast tumors (Lu et al., 2024), or even the complete inhibition of EGFR/PI3K/AKT and mTOR mediated pathways using natural compounds (Ganesan et al., 2024). In our resistance model, we hypothesize that the pharmacological inhibition of KRAS, coupled with the development of resistance, may have accentuated the proliferative signaling pathway mediated by PI3K-AKT-mTOR. This pathway is recognized for its compensatory actions in response to such inhibitory interventions (Ersahin et al., 2015). In patients with advanced-stage KRAS^G12C^ lung adenocarcinoma treated with sotorasib for 17 weeks revealed an initial response followed by acquired resistance. In these cases, activation of the MAPK pathway, AKT and mTOR signaling was detected in almost all samples post-sotorasib treatment, with no supplementary mutations identified capable of reactivating the KRAS and MAPK signaling axis (Tsai et al., 2022), similar to that observed in our cellular sotorasib-resistance model. A recent investigation highlights that the pharmacological inhibition of the PI3K-mediated signaling axis can effectively restore sensitivity in KRAS^G12C^ mutant cell lines that exhibit intrinsic resistance to sotorasib (Qi et al., 2023).

Considering the increase in pAKT isoforms, accompanied by pAKT immunostaining and upregulation of genes implicated in AKT signaling within our sotorasib-resistant cellular model, we initiated a therapeutic combination study involving sotorasib and MK2206, an AKT inhibitor. The outcomes revealed notable inhibition scores, disclosing robust synergistic activity between the drugs, even at low combination doses.

To evaluate the efficacy of AKT inhibition as a therapeutic strategy for restoring sotorasib sensitivity in resistant cells, we employed a similar experimental approach used for the anti-p38 therapy. Surprisingly, our findings indicated that both pharmacological and genetic inhibition of AKT effectively restored sensitivity to sotorasib. This was evidenced by a substantial reduction in cell viability and a significant increase in apoptosis under the experimental conditions. Moreover, AKT inhibition demonstrated a more potent effect compared to p38 inhibition-based therapy. Consequently, AKT signaling appears to be more critical for preserving cellular replicative capacity than p38 signaling. Nevertheless, based on the aforementioned results, it is uncertain whether a combinatorial approach employing both therapies would yield a synergistic effect, surpassing the efficacy of each individual treatment.

Collectively, these findings highlight the significance of adaptive mechanisms in sotorasib resistance in NSCLC cancer cell, emphasizing the need consideration of combination therapies involving other approved AKT and p38 inhibitors, able of restoring KRAS^G12C^ cells sensibility to sotorasib. Conclusively, our findings uncovered non-genetic mechanisms underlying EMT activation and its role in acquired resistance to sotorasib. This discovery has the potential to inform the design of novel clinical trials and promising therapeutic strategies to overcome this resistance, ultimately improving patient outcomes.

## Data Availability

The original contributions presented in the study are publicly available. This data can be found here: Gene Expression Omnibus (GEO), accession numbers GSE204752 and GSE204753.
