# Supplementary material for: Sotorasib resistance triggers epithelial-mesenchymal transition and activates AKT and P38-mediated signaling
Source: Front Mol Biosci. 2025 Jan 30;12:1537523. doi: 10.3389/fmolb.2025.1537523 (PMC11821485; doi:10.3389/fmolb.2025.1537523)
Supplement: Supplementary file 1 [file Supplementaryfile1.docx]

***Original research***

**Sotorasib resistance triggers epithelial-mesenchymal transition and activates AKT and P38-mediated signaling**

Raquel Arantes Megid^1^; Guilherme Gomes Ribeiro^1^; Izabela Natalia Faria Gomes^1^; Ana Carolina Laus^1^; Letícia Ferro Leal^1^; Luciane Sussuchi da Silva^1^; Abu-Bakr Adetayo Ariwoola; Josiane Mourão Dias^2^; Rui Manuel Reis^1,3,4^ Renato José da Silva-Oliveira^1,5*^

**Additional i information**

**Supplementary table 1.**

Primary and secondary antibodies utilized for Western Blot and Reverse Phase Protein Arrays (RPPA) analysis.

| Anti-body name | Type | Phosphorylation site. | Purchased from | Cat. Number | RRID |
| --- | --- | --- | --- | --- | --- |
| KRAS | Primary |  | Abcam Plc, Inc | #ab108602 | AB_10891004 |
| pEGFR | Primary | Tyr1068 | Cell signaling | #3777 | AB_2096270 |
| tEGFR | Primary |  | Cell signaling | #4267 | AB_2246311 |
| pErBb-2 | Primary | Tyr1221/1222 | Cell signaling | #2243 | AB_490899 |
| tErbB-2 | Primary |  | Cell signaling | #4290 | AB_10557104 |
| pErbB-3 | Primary | Tyr1289 | Cell signaling | #2842 | AB_11178795 |
| pErbB-4 | Primary | Tyr1284 | Cell signaling | #4757 | AB_2099987 |
| pP38MAPK | Primary | Thr180/Tyr182 | Cell signaling | #4511 | AB_2139682 |
| tP38 MAPK | Primary |  | Cell signaling | #8690 | AB_10999090 |
| pP44/42(Erk1/2) | Primary | Thr202/Tyr204 | Cell signaling | #4370 | AB_2315112 |
| t44/42 MAPK (Erk1/2) | Primary |  | Cell signaling | #4695 | AB_390779 |
| pAKT | Primary | Ser473 | Cell signaling | #4060 | AB_2315049 |
| AKT(pan) | Primary |  | Cell signaling | #4691 | AB_915783 |
| AKT1 | Primary |  | Cell signaling | #2938 | AB_915788 |
| AKT2 | Primary |  | Cell signaling | #3063 | AB_2225186 |
| AKT3 | Primary |  | Cell signaling | #4059 | AB_2225351 |
| β-actin | Primary |  | Cell signaling | #3700 | AB_2242334 |
| Anti-rabbit | Secondary |  | Cell signaling | #7074 | AB_2099233 |
| Anti-mouse | Secondary |  | Cell signaling | #7076 | AB_330924 |

**Supplementary table 2.**

Genes analyzed by real time PCR.

| Gene | Primer Foward (5' - 3') | Primer Reverse (5' - 3') | Size (bp) | Annealing temperature °C |
| --- | --- | --- | --- | --- |
| β-actin | GGACTTCGAGCAAGAGATGG | AGCACTGTGTTGGCGTACAG | 234 | 63 |
| Snail | CTCTAGGCCCTGGCTGCTAC | TGACATCTGAGTGGGTCTGG | 134 | 63 |
| Slug | CTTTTTCTTGCCCTCACTGC | ACAGCAGCCAGATTCCTCAT | 161 | 63 |
| E-cadherin | TGCCCAGAAAATGAAAAAGG | GTGTATGTGGCAATGCGTTC | 200 | 61 |
| N-cadherin | ACAGTGGCCACCTACAAAGG | TGATCCCTCAGGAACTGTCC | 392 | 64 |
| Vimentin | GGGACCTCTACGAGGAGGAG | AAGATTGCAGGGTGTTTTCG | 177 | 63 |
| MMP9 | GCACGACGTCTTCCAGTACC | CAGGATGTCATAGGTCACGTAGC | 124 | 59 |
| MMP 14 | CACTGCCTACGAGAGGAAGG | TCCCTTCCCAGACTTTGATG | 269 | 63 |
| MMP 24 | TGAAGGCATTGACACAGCTC | CGCTCAGTTTCTGGTTGTCA | 242 | 63 |

**Abbreviations:** EMC, Epithelial-mesenchymal transition; BP, base pair

**Supplementary table 3.**

Values of IC_50_ calculated after clone isolation of sotorasib-resistant.

| Cell Line / clones | IC_50_ (nM) |
| --- | --- |
| H358 Parental | 4.02 ± 0.1 |
| H358 Clone A1 | >1000 |
| H358 Clone A2 | 971.8 ± 17.1 |
| H358 Clone B1 | >1000 |
| H358 Clone E1 | 665.5 ± 9.3 |
| H358 Clone H1 | >1000 |
| H358 Clone H4 | >1000 |

**Supplementary table 4.**

Differentially expressed genes involved in H358-R sotorasib resistant cell lines, using the nCounter PanCancer Pathways.

| Gene | Description | *Fold change* | *p valor* |
| --- | --- | --- | --- |
| *down-regulated genes* | | | |
| *ANGPT1* | *angiopoietin 1* | -3,742 | 0,010 |
| *CD19* | *CD19 molecule* | -2,983 | 0,008 |
| *NOS3* | *nitric oxide synthase 3 (endothelial cell)* | -2,367 | 0,012 |
| *TGFB2* | *transforming growth factor_ beta 2* | -2,105 | 0,005 |
| *CCNB1* | *cyclin B1* | -2,063 | 0,009 |
| *DDIT3* | *DNA-damage-inducible transcript 3* | -1,835 | 0,022 |
| *POLE2* | *polymerase (DNA directed)_ epsilon 2_ accessory subunit* | -1,597 | 0,002 |
| *PTTG2* | *pituitary tumor-transforming 2* | -1,535 | 0,013 |
| *GADD45B* | *growth arrest and DNA-damage-inducible_ beta* | -1,535 | 0,002 |
| *BMP2* | *bone morphogenetic protein 2* | -1,518 | 0,002 |
| *INHBB* | *inhibin_ beta B* | -1,516 | 0,001 |
| *up-regulated genes* | | | |
| *ARID1B* | *AT rich interactive domain 1B (SWI1-like)* | 1,534 | 0,004 |
| *CDKN2B* | *cyclin-dependent kinase inhibitor 2B (p15_ inhibits CDK4)* | 1,554 | 0,008 |
| *NOTCH3* | *notch 3* | 1,584 | 0,011 |
| *CDKN1A* | *cyclin-dependent kinase inhibitor 1A (p21_ Cip1)* | 1,595 | 0,041 |
| *MLLT4* | *myeloid/lymphoid or mixed-lineage leukemia; translocated to_ 4* | 1,617 | 0,000 |
| *SPP1* | *secreted phosphoprotein 1* | 1,639 | 0,043 |
| *KITLG* | *KIT ligand* | 1,666 | 0,006 |
| *PLCG2* | *phospholipase C_ gamma 2 (phosphatidylinositol-specific)* | 1,690 | 0,013 |
| *PLA2G4A* | *phospholipase A2_ group IVA (cytosolic_ calcium-dependent)* | 1,698 | 0,003 |
| *MAP3K5* | *mitogen-activated protein kinase kinase kinase 5* | 1,717 | 0,027 |
| *FGFR3* | *fibroblast growth factor receptor 3* | 1,725 | 0,023 |
| *IL1RAP* | *interleukin 1 receptor accessory protein* | 1,726 | 0,020 |
| *COL1A1* | *collagen_ type I_ alpha 1* | 1,732 | 0,049 |
| *HDAC4* | *histone deacetylase 4* | 1,738 | 0,020 |
| *MAP3K8* | *mitogen-activated protein kinase kinase kinase 8* | 1,748 | 0,006 |
| *IL1R1* | *interleukin 1 receptor_ type I* | 1,834 | 0,042 |
| *PRDM1* | *PR domain containing 1_ with ZNF domain* | 1,929 | 0,028 |
| *ITGA2* | *integrin_ alpha 2 (CD49B_ alpha 2 subunit of VLA-2 receptor)* | 1,963 | 0,001 |
| *GLI3* | *GLI family zinc finger 3* | 2,025 | 0,039 |
| *NR4A1* | *nuclear receptor subfamily 4_ group A_ member 1* | 2,171 | 0,044 |
| *PTCH1* | *patched 1* | 2,291 | 0,026 |
| *SHC4* | *SHC (Src homology 2 domain containing) family_ member 4* | 2,627 | 0,015 |
| *IL1A* | *interleukin 1_ alpha* | 2,788 | 0,016 |
| *FOXO4* | *forkhead box O4* | 2,875 | 0,014 |
| *BIRC3* | *baculoviral IAP repeat containing 3* | 2,939 | 0,010 |
| *LEF1* | *lymphoid enhancer-binding factor 1* | 3,091 | 0,002 |
| *RASGRP1* | *RAS guanyl releasing protein 1 (calcium and DAG-regulated)* | 3,143 | 0,032 |
| *PITX2* | *paired-like homeodomain 2* | 3,913 | 0,049 |
| *IL20RA* | *interleukin 20 receptor_ alpha* | 3,929 | 0,013 |
| *WNT2B* | *wingless-type MMTV integration site family_ member 2B* | 4,857 | 0,025 |

**Supplementary table 5.**

Synergistic score estimate using ZIP, Loewe and Bliss method.

| ﻿Drug combination | Synergy score | Most synergistic area score | Method |
| --- | --- | --- | --- |
| Adezmapimod + Sotorasib | ﻿-2.33 | ﻿-0.75 | ZIP |
| Afatinib | ﻿-14.22 | ﻿-5.26 | ZIP |
| MK2206 | ﻿1.50 | ﻿2.74 | ZIP |
| ﻿Drug combination | **Synergy score** | **Most synergistic area score** | **Method** |
| Adezmapimod + Sotorasib | -1.75 | -0.18 | Loewe |
| Afatinib | -0.91 | 2.91 | Loewe |
| MK2206 | -0.57 | 0.90 | Loewe |
| ﻿Drug combination | **Synergy score** | **Most synergistic area score** | **Method** |
| Adezmapimod + Sotorasib | ﻿-3.73 | ﻿-1.58 | Bliss |
| Afatinib | ﻿-16.87 | ﻿-3.08 | Bliss |
| MK2206 | ﻿0.33 | ﻿1.69 | Bliss |

**Supplementary figure 1.**

Densitometry analyses from reverse protein phase arrays


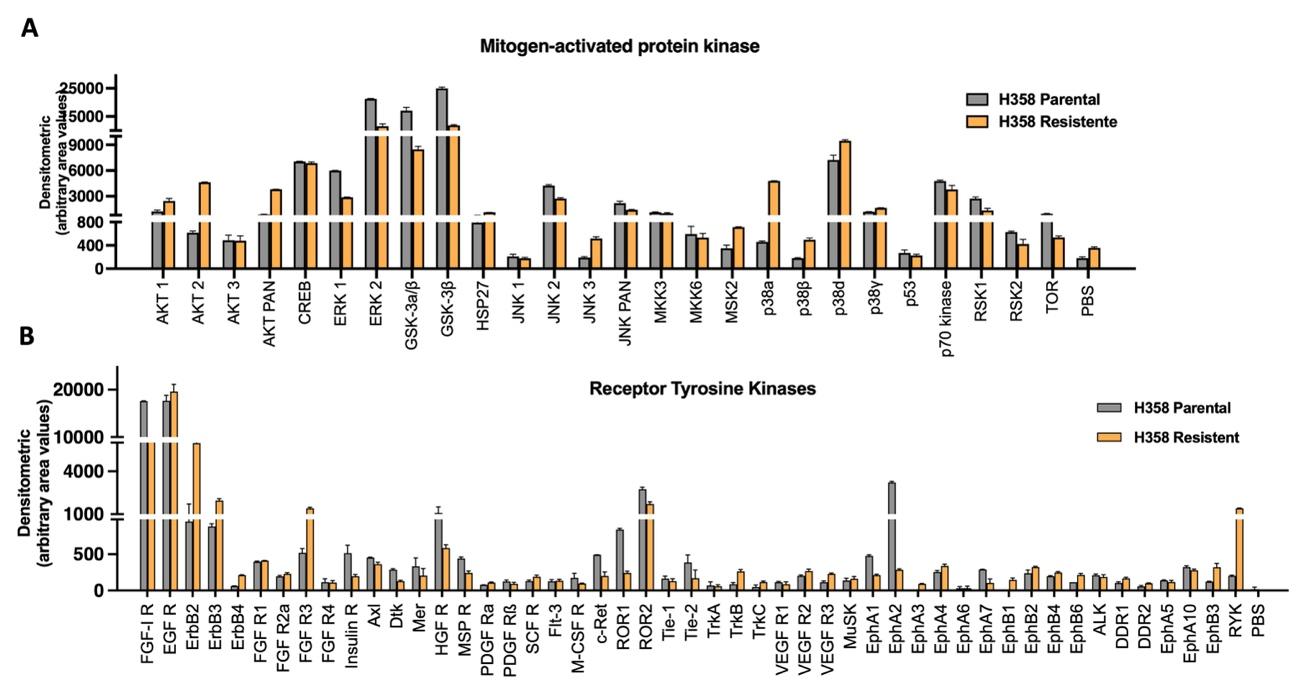


Fig. S1. Densitometry analyses from reverse protein phase arrays. (A) Mitogen-activated protein kinase (MAPK) array of H358 parental and H358-R sotorasib resistant cell lines; (B) Receptor Tyrosine Kinases (RTK) array of H358 parental and H358-R sotorasi(B) Receptor Tyrosine Kinases (RTK) array of H358 parental and H358-R sotorasib-resistant cell lines. Bars graphics represent the arbitrary densitometry area.

**Supplementary figure 2.**

Immunohistochemical staining


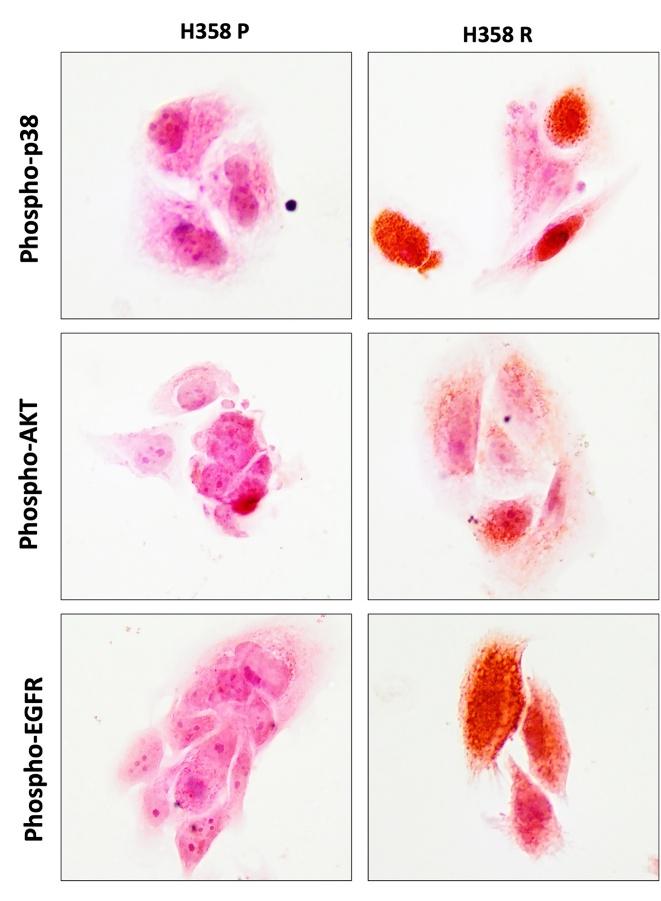


Fig. S2. Photomicrography at 100× magnification from immunohistochemical staining for comparative phosphorylated protein expression among the H358-P parental and H358-R sotorasib-resistant cell lines.

**Supplementary figure 3.**

Confirmation of pAKT and p38 knockdown


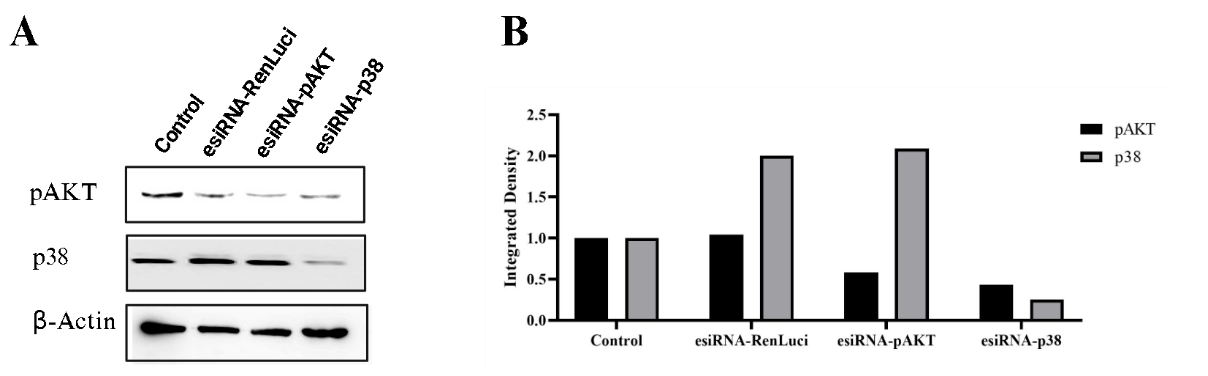


Fig. S3. Confirmation of p38 and pATK gene knockdown via RNA interference. (A) Western blot analysis of total and phosphorylated p38 and pATK proteins after silencing. (B) Densitometry quantification of total and phosphorylated p38 and pATK protein level

**Supplementary methodology information regarding the NanoString painel analysis**

Raw NanoString data was pre-processed using nSolver v4.0® and normalized by housekeeping genes with the NanoStringNorm v1.2.1.1 package in R v3.6.3. Log2-transformed expression values were used for differential expression analysis (FC ≥ ±1.5, p < 0.05). Differentially expressed genes were visualized as a heatmap with hierarchical clustering using ComplexHeatmap v2.0.0. STRING v11.5 and ShinyGO were used for in silico network and enrichment analyses, respectively.
